# High expression of microRNA-126 relates to favorable prognosis for colon cancer patients

**DOI:** 10.1038/s41598-021-87985-3

**Published:** 2021-05-05

**Authors:** Hallgeir Selven, Lill-Tove Rasmussen Busund, Sigve Andersen, Roy M. Bremnes, Thomas Karsten Kilvær

**Affiliations:** 1 Department of Oncology, University Hospital of North Norway, 9038 Tromsö, Norway; 2 Department of Clinical Medicine, UiT The Arctic University of Norway, Tromsö, Norway; 3 Department of Medical Biology, UiT The Arctic University of Norway, Tromsö, Norway; 4 Department of Clinical Pathology, University Hospital of North Norway, Tromsö, Norway

**Keywords:** Oncology, Gastrointestinal cancer

## Abstract

miR-126 has been identified both as a tumor suppressor and an oncogene in different types of cancer. The aim of this study was to investigate the prognostic impact of miR-126-expression in colon cancer patients. Tumor tissue from 452 patients operated for stage I–III colon cancer was retrospectively collected and tissue microarrays were constructed. miR-126 expression was evaluated by in situ hybridization and analyzed using digital pathology. To isolate the compartment specific contribution of miR-126, tumor and adjacent tumor stroma were considered separately. In univariate analyses, high expression of miR-126 in tumor and stroma was related to increased disease-specific survival (p < 0.001 and p = 0.005, respectively). In multivariate analyses, high miR-126 expression in tumor remained a significant independent predictor of improved disease-specific survival (HR = 0.42, CI 0.23–0.75, p = 0.004). Within different TNM-stages there was a tendency towards the same results, but with statistically significant results in stage II only (p = 0.007). High expression of miR-126 is an independent positive prognostic factor in stage I–III colon cancer. This finding may be used to identify patients in need of adjuvant chemotherapy.

## Introduction

In the US, colon cancer is the 4^th^ most common cancer among women and men, separately and combined. Estimates show that more than 100,000 patients will be diagnosed with colon cancer in the US in 2020. Moreover, the estimated life time risk of contracting colon cancer in the US is 4%^1^. Globally, colon cancer is the 4^th^ most common cancer type, and the 5^th^ leading cause of cancer-related death^2^.

Prognostication of colon cancer patients relies on the TNM-system and histopathological criteria according to the American Joint Committee on Cancer (AJCC) and the Union for International Cancer Control (UICC)^3^. Clinical management, including the need for adjuvant treatment, is assessed according to the TNM staging system^4^. However, contrary to other forms of cancer, the TNM staging system for colon cancer is rather imprecise, and the risk of recurrence varies significantly for patients within the same pathological stage^5^. This lack of precision has encouraged researchers to search for more precise prognostic and predictive biomarkers.

MicroRNAs (miRNAs or miRs), first identified in 1993, are single-stranded, non-coding RNAs, approximately 22 nucleotides long, regulating gene expression at the post-transcriptional level. Many miRNAs exist in the human genome, and each miRNA can potentially regulate hundreds of mRNAs, making them important mediators of cellular processes (differentiation, proliferation, apoptosis, stress response etc.). In cancer, miRNAs regulate molecular pathways by targeting oncogenes and tumor suppressors. Moreover, they play significant roles in cancer-stem-cell biology, angiogenesis, epithelial-mesenchymal-transition, metastasis and drug resistance, among others^6,7^.

miR-126 mediates developmental angiogenesis in vivo and is the most highly enriched miR in endothelial cells. It represses intracellular inhibitors of angiogenic signaling (VEGF-pathway), thus enhancing the pro-angiogenic actions of VEGF and FGF, leading to blood-vessel formation. In zebrafish, knockdown of miR-126, results in loss of vascular integrity and hemorrhage during embryonic development^8,9^.

miR-126 is aberrantly expressed in most cancer types, including cancers of the gastrointestinal tract, genital tracts, breast cancer, thyroid cancer, lung cancer and acute myeloid leukemia^10^. Previous studies have shown a loss of miR-126 expression in colon cancer cell lines compared to normal colon epithelium. Reconstitution of miR-126 results in significant growth reduction^11^. Hence, miR-126 is considered a suppressor of colon cancer development.

Further, corroborating the results from cell line studies, Hansen et al. demonstrated that patients with metastatic colorectal cancer (mCRC) responding to 1st line chemotherapy presented with a significantly higher median miR-126 expression in tumor associated vasculature *vs* non-responders. This translated to a significantly enhanced median progression-free survival (PFS) (11.5 months *vs* 6.0 months) for patients with high *vs* low miR-126 expressing tumors^12^. In patients operated for stage II colon cancer, low expression of miR-126 correlated to established, negative prognostic factors (T4 and high malignancy grade among others). Patients with high miR-126 expression had a significantly improved overall survival compared to patients with low miR-126 expression^13^.

Other studies have shown that miR-126 is detectable in plasma, and that an increase in plasma miR-126 may be predictive of tumor response in colon cancer patients receiving palliative chemotherapy^14^. These factors may provide an attractive, non-invasive method to evaluate treatment response.

Several studies have shown high miR-126 expression to be a positive prognostic factor in colon cancer patients. But most of these studies used qPCR, excluding the possibilities to detect specific expression in the various tumor compartments. We sought to explore the prognostic impact of miR-126 expression in both the tumor epithelial and surrounding stromal cells utilizing in situ hybridization. The study was conducted on primary tumors from 452 stage I–III colon cancer patients. We hypothesized that miR-126 is a clinically relevant biomarker for this group of patients.

## Results

### Patient characteristics

A total number of 452 patients were included in this study. The main patient characteristics are summarized in Table 1. Median age at surgery was 74 years (range 30–94), median follow-up of survivors was 173 months (range 119–239). There were 243 females (53.8%) and 209 males (46.2%). Median tumor size was 50 mm (range 10–180 mm). Nine (2%), 76 (16.8%), 320 (70.8%) and 47 (10.4%) patients were categorized as pT1-4, respectively. Lymph node-positive disease was present in 161 patients (35.6%). According to the pTNM-stage, 72 (15.9%), 219 (48.5%) and 161 (35.6%) patients were diagnosed with stage I-III disease, respectively. A total of 87 patients (19.2%) received adjuvant chemotherapy. Nordic-FLv (5-FU/Leukovorin bolus schedule) was the chosen regimen until 2004 when it was shown that adding Oxaliplatin gave superior results for patients < 70 years. From 2004, patients < 70 years were consequently offered treatment with Nordic FLOX (Oxaliplatin/5-FU/Leukovorin bolus schedule)^15–17^. Thus, 69 patients (79.3%) were given Nordic-FLv and five patients (5.7%) Nordic FLOX. Eleven patients (12.6%) were initially administered Nordic FLOX, but were later converted to Nordic-FLv because of unacceptable toxicity. Two patients (2.3%) were given adjuvant radio-/chemotherapy. At the end of follow-up, 119 patients had verified recurrent disease (26.3%) and 313 (69.2%) were dead, either due to colon cancer (108, 34.5%) or other causes (205, 65.5%).

**Table 1:** Frequency table summarizing the median and range and total number of important continuous and cathegorical clinicopathological variables, respectively.

Variable	n (%)	5-y OS
**Age at diagnosis**		
Median	74.0 years (30–94 years)	N/A
**Tumor size**		
Median	50 mm (10–180 mm)	N/A
Missing	2	N/A
**Gender**		
Male	209 (46.2%)	59.8%
Female	243 (53.8%)	62.1%
**Relapse**		
No	333 (73.7%)	74.5%
Yes	119 (26.3%)	23.5%
**pT status**		
T1	9 (2.0%)	88.9%
T2	76 (16.8%)	65.8%
T3	320 (70.8%)	61.6%
T4a	26 (5.8%)	46.2%
T4b	21 (4.6%)	42.9%
**pN status**		
N0	291 (64.4%)	68.4%
N1a	56 (12.4%)	50.0%
N1b	55 (12.2%)	52.7%
N1c	1 (0.2%)	0%
N2a	27 (6.0%)	40.7%
N2b	22 (4.9%)	40.9%
**pTNM stage (groups)**		
I	72 (15.9%)	70.8%
II	219 (48.5%)	67.6%
III	161 (35.6%)	47.8%
**Differentiation**		
Well	36 (8.0%)	80.6%
Moderate	329 (72.8%)	59.6%
Poor	75 (16.6%)	60.0%
Undifferentiated	4 (0.9%)	25.0%
Missing	8 (1.8%)	N/A
**Site**		
Right	227 (50.2%)	63.0%
Transverse	65 (14.4%)	49.2%
Left	21 (4.6%)	66.7%
Sigmoid	136 (30.1%)	61.8%
Missing	3 (0.7%)	N/A
**Weight loss**		
< 10%	248 (54.9%)	68.5%
≥ 10%	94 (20.8%)	46.8%
Missing	110 (24.3%)	N/A
**Performance status (ECOG)**		
0	237 (52.4%)	70.0%
1	149 (33.0%)	58.4%
2	54 (11.9%)	33.3%
3	8 (1.8%)	25.0%
Missing	4 (0.9%)	N/A
**Adjuvant chemotherapy**		
No	365 (80.8%)	60.3%
Yes	87 (19.2%)	67.8%
**Postoperative complications**		
No	358 (79.2%)	64.5%
Yes	94 (20.8%)	47.9%
**miR-126 expression tumor**		
High	140 (31.0%)	67.9%
Low	300 (66.4%)	58.0%
Missing	12 (2.7%)	N/A
**miR-126 expression stroma**		
High	109 (24.1%)	63.3%
Low	341 (75.4%)	60.4%
Missing	2 (0.4%)	N/A

For categorical variables five-year overall survival is given in percent.

### Expression of miR-126 and its correlations

**Figure 1:**
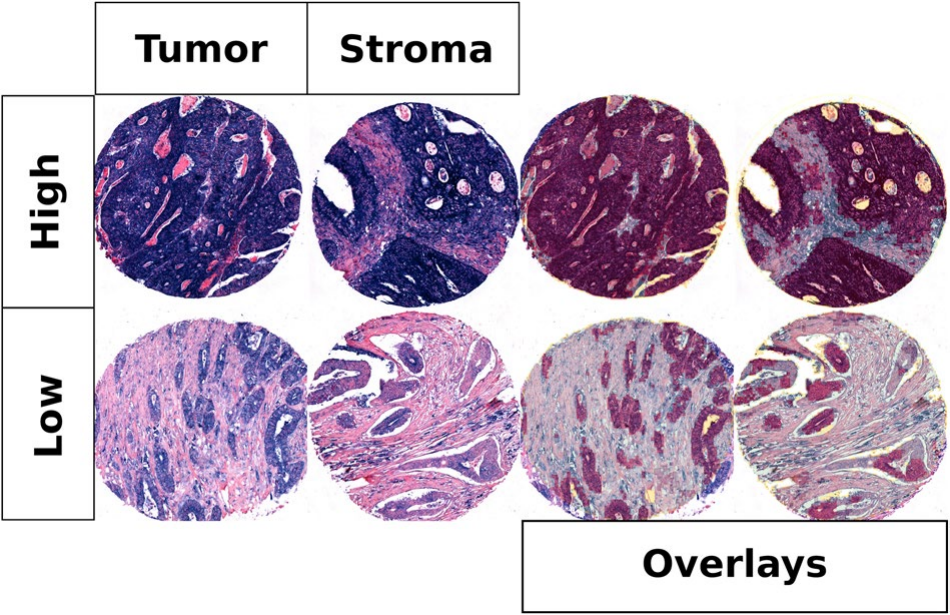
High and low scores for miR-126 in tumor and stroma with and without overlays.

miR-126 was expressed in tumor epithelial cells as well as stromal cells including spindle shaped cells (likely fibroblasts, endothelial cells and vascular smooth muscle cells) and immune cells (Fig. 1). miR-126 expression in tumor and stroma was highly correlated (r = 0.60). Table 2B shows associations between miR-126-expression in both tumor and stroma and clinicopathological variables. Low miR-126-expression in stroma was significantly associated with increasing pStage (p = 0.027) as well as histological grade (p = 0.004).

**Table 2:** (A) Clinicopathological variables and miR-126 in tumor and stroma as predictors of disease-specific survival for colon cancer patients (univariate analyses, log-rank test, n = 452), (B) dichotomized miR-126 in tumor and stroma and their distribution over and correlation with clinicopathological variables (chi-square and Fisher’s exact tests).

	A					B					
	N (%)	5 Year	Median	HR (95%CI)		miR126 in tumor			miR126 in stroma		
					P	Low	High		Low	High	P
**Age**					0.013			0.358			0.130
≤ 65	110 (24)	88	NA	1.00		78	30		89	20	
> 65	342 (76)	76	NA	1.83 (1.21–2.77)		222	110		252	89	
**Gender**					0.388			0.751			1.000
Female	243 (54)	78	NA	1.00		165	74		184	59	
Male	209 (46)	81	NA	0.85 (0.58–1.23)		135	66		157	50	
**Weight_loss**					0.012			0.092			0.621
< 10%	248 (55)	82	NA	1.00		157	83		192	55	
≥ 10%	94 (21)	68	NA	1.74 (1.06–2.87)		69	22		70	24	
Missing	110 (24)										
**ECOG_status**					0.424			0.997			0.993
0	237 (52)	82	NA	1.00		157	75		178	57	
1	149 (33)	76	NA	1.31 (0.86–1.99)		98	45		113	36	
2	54 (12)	74	NA	1.18 (0.62–2.28)		36	17		40	14	
3	8 (2)	50	47	2.31 (0.31–16.98)		6	2		6	2	
Missing	4 (1)										
**Site**					0.951			0.824			0.700
Sigmoid	227 (50)	79	NA	1.00		152	69		171	56	
Transversum	65 (14)	78	NA	0.94 (0.52–1.68)		45	19		47	18	
Left	21 (5)	80	NA	1.15 (0.47–2.78)		14	5		18	3	
Right	136 (30)	79	NA	1.09 (0.71–1.67)		87	46		102	32	
Missing	3 (1)										
**pStage**					< 0.001			0.388			0.027
1	72 (16)	94	NA	1.00		46	25		53	19	
2	219 (48)	89	NA	2.27 (1.35–3.81)		142	72		156	63	
3	161 (36)	59	NA	8.04 (4.58–14.11)		112	43		132	27	
**Differentiation**					< 0.001			0.311			0.006
Well	36 (8)	89	NA	1.00		19	16		27	9	
Moderate	329 (73)	78	NA	1.47 (0.76–2.82)		224	98		259	68	
Poor	75 (17)	79	NA	1.45 (0.67–3.15)		49	22		48	27	
Undifferentiated	4 (1)	25	10	11.34 (0.45–283.15)		3	1		1	3	
Missing	8 (2)										
**Vasc+**					< 0.001			0.305			0.575
No	199 (44)	83	NA	1.00		121	69		151	48	
Yes	19 (4)	45	30	5.22 (1.66–16.37)		14	4		15	3	
Missing	234 (52)										
**Resection margins**					0.005			0.519			0.630
0 mm	31 (7)	54	68	1.00		23	6		26	5	
< 1 mm	42 (9)	70	NA	0.56 (0.2–1.54)		31	11		31	11	
1–2 mm	35 (8)	88	NA	0.3 (0.11–0.85)		21	14		25	10	
2–10 mm	121 (27)	85	NA	0.33 (0.14–0.77)		81	35		94	26	
10–50 mm	155 (34)	80	NA	0.4 (0.17–0.95)		100	50		112	43	
> 50 mm	46 (10)	80	NA	0.3 (0.11–0.81)		29	17		36	9	
Missing	22 (5)										
**miR126 in tumor**					< 0.001						
Low	300 (66)	75	NA	1.00							
High	140 (31)	88	NA	0.43 (0.29–0.64)							
Missing	12 (3)										
**miR126 in stroma**					0.005						
Low	341 (75)	76	NA	1.00							
High	109 (24)	88	NA	0.46 (0.29–0.71)							
Missing	2 (0)										

ECOG, eastern cooperative oncology group.

### Univariate analysis

**Figure 2:**
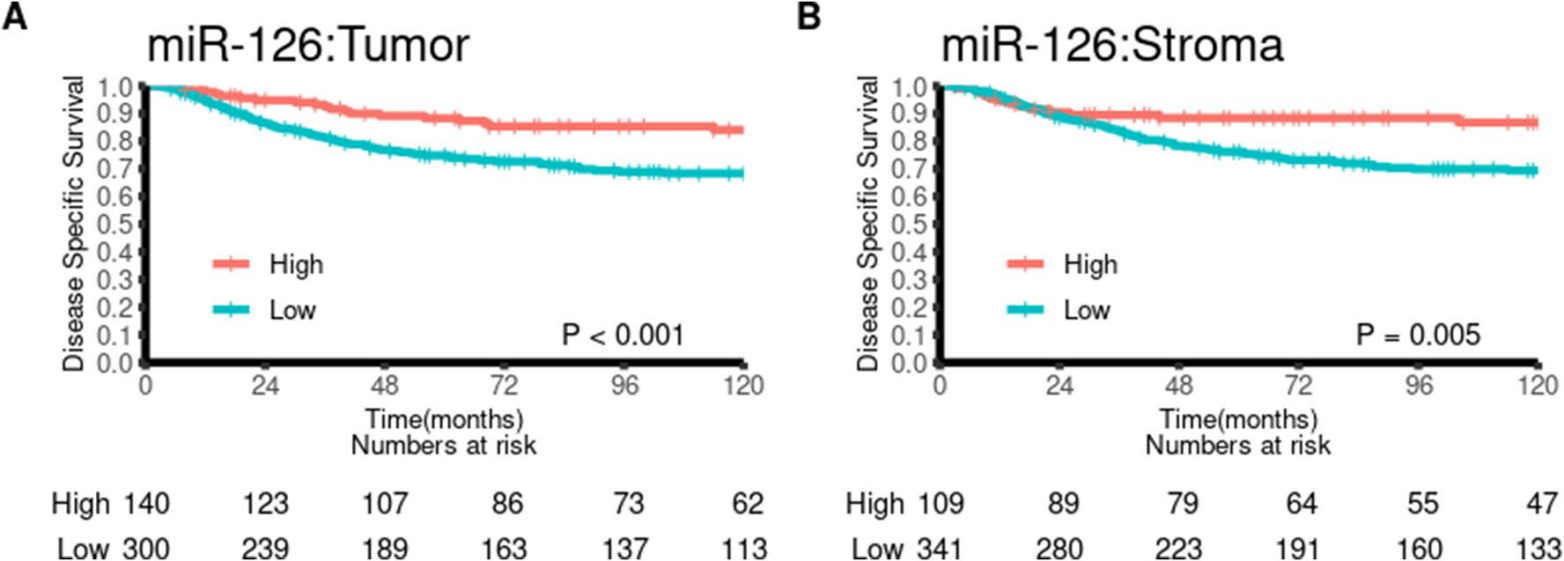
Disease-specific survival curves for tumor (**A**) and stromal (**B**) expression of miR-126, using the optimal cut-offs for each marker.

Univariate survival analyses according to clinicopathological variables and miR-126 are summarized in Table 2A and visualized in Fig. 2. Age (p = 0.013), weight loss (p = 0.012), pathological stage (p < 0.001), histological grade (p < 0.001), vascular infiltration (p < 0.001), resection margins (p = 0.005), miR-126 expression in tumor (p < 0.001) and miR-126 expression in stroma (p = 0.005) were all significant indicators of DSS in the total population.

Analyses in subgroups according to pTNM stage, revealed that high expression of miR-126 in both tumor and stroma showed a tendency towards being a positive predictor of DSS within each TNM stage (S1 Fig). This effect was significant in stage II patients (p = 0.007 and 0.009 for tumor and stroma, respectively).

### Multivariate analysis

Multivariate analyses are summarized in Table 3. Age (HR 1.03, CI95% 1.01–1.05, p = 0.002) and miR-126-expression in tumor (HR 0.45, CI 0.27–0.76, p = 0.002) were independently associated with DSS. Histological grade also showed statistical significance overall (p = 0.032), but with non-significant results within the subgroups (moderate (HR 0.76, CI 0.35–1.68, p = 0.5), poor (HR 0.63, CI 0.26–1.57, p = 0.322), or undifferentiated (HR 4.02, CI 0.99–16.36, p = 0.052)). Pathological stage was statistically significant overall (p < 0.001), but in subgroups only stage III was statistically significant (stage II: HR 2.12, CI 0.82–5.53, p = 0.123, stage III: HR 7.35, CI 2.89–18.72, p < 0.001).

**Table 3:** Multivariate models for miR-126 in (A) tumor and (B) stroma (cox proportional hazards test, n = 452).

	(A)		95.0% CI		(B)		95.0% CI	
	p	HR	Lower	Upper	p	HR	Lower	Upper
Age at diagnosis	**0.002**	1.03	1.01	1.05	**0.003**	1.03	1.01	1.05
**Differentiation**	**0.032**				**0.027**			
Well differentiated		1.00				1.00		
Moderately differentiated	0.500	0.76	0.35	1.68	0.706	0.86	0.39	1.89
Poorly differentiated	0.322	0.63	0.26	1.57	0.653	0.81	0.33	2.01
Undifferentiated	0.052	4.02	0.99	16.36	**0.020**	5.27	1.30	21.46
**pTNM**	** < 0.001**				** < 0.001**			
pTNM-stage I		1.00				1.00		
pTNM-stage II	0.123	2.12	0.82	5.53	0.122	2.12	0.82	5.51
pTNM-stage III	** < 0.001**	7.35	2.89	18.72	** < 0.001**	6.76	2.66	17.18
**miR-126 tumor**								
Low		1.00						
High	**0.002**	0.46	0.27	0.76				
**miR-126 stroma**								
Low						1.00		
High					**0.049**	0.55	0.30	1.00

Statistically significant results indicated in bold.

## Discussion

In the presented material, DSS was significantly associated with previously well-known prognostic factors including age, weight loss, pathological stage, histological grade, vascular infiltration and resection margins^18^. In addition, low expression of miR-126 in both tumor and stroma was significantly associated with shorter DSS. Overall, patients with high expression of miR-126 in cancer cells had a 5-year DSS of 88% compared with 75% for low expression. Likewise, patients with high expression of miR-126 in tumor stroma had a 5-year DSS of 88% compared with 76% for low expression. To our knowledge, this is the first paper to demonstrate compartment-specific prognostic impact of miR-126 in cancer and stromal cells using in situ hybridization in a large, homogenous cohort of primary tumors from colon cancer patients treated with curative intent. Moreover, we introduce an open source pipeline for miR expression analyses that may be automated and adapted to other use cases.

A meta-analysis, published in 2016, assessed the effectiveness of miR-126 as a prognostic biomarker for various cancers^19^. In small cell lung cancer, miR-126 overexpression induced delayed G1 phase of the cancer cell cycle. Likewise, in breast cancer cells, cell cycle progression was inhibited from the G1/G0 phase to S phase by miR-126. In pancreatic cancer cells, low expression of miR-126 led to increased epithelial-mesenchymal transition, a process involved in metastasis. In some gastric cancer cell lines, however, overexpression of miR-126 resulted in tumor growth by regulating its downstream target genes. According to this meta-analysis, higher miR-126 expression predicted better OS in digestive system and respiratory system cancers.

miR-126 normally functions as a growth suppressor in colon cells. In colon cancer cell lines, miR-126 is frequently downregulated compared to normal colon epithelium. Guo et al. showed that miR-126 regulates phosphatidylinositol 3-kinase (PI3K)-signaling by targeting the PI3K regulatory subunit beta (p85β) through translational repression^11^. Downregulation of miR-126 leads to upregulation of PI3K-signalling, resulting in tumor growth. Restoration of miR-126 yielded a marked reduction in p85β. Chemokine receptor 4 (CXCR4) is highly expressed in various types of cancer, and is considered important for mobilization, migration, proliferation and survival of different cell types. CXCR4 is a target for miR-126-mediated repression, and this repression may inhibit migration and invasion of colon cancer cells. The Ras homolog gene family, member A (RhoA), is associated with invasion and poor prognosis in colorectal cancer. Rho function by downstream signaling via PI3K and ROCK (Rho-associated coiled-coil-containing protein kinase) among others. miR-126 has been shown to act as a tumor suppressor via RhoA/ROCK inhibition in cancer cells. Yuan et al. demonstrated that colon cancer cell invasion and migration was inhibited by miR-126, in vivo and in vitro, by down-regulating CXCR4 and inactivating the RhoA signaling pathway^20^.

In 2014, Liu et al. published an article on the prognostic impact of miR-126 expression in colorectal cancer with similar results to ours. By using qPCR they found approximately the same DSS in patients with high expression of miR-126, but even worse prognosis for patients with low expression of miR-126^21^. A possible explanation for this worse outcome could be the inclusion of patients with metastatic disease in their study, while we included only patients treated with curative intent.

Hansen et al. assessed the prognostic value of miR-126 and microvessel density (MVD) in patients with stage II colon cancer^13^. Their study included 560 patients, and the primary endpoints were recurrence-free cancer specific survival (RF-CSS) and overall survival (OS). qPCR was performed to analyze miR-126 expression. Like in our study, they found that low expression of miR-126 correlated to histological grade and a worse OS (p = 0.03). In their multivariate analysis, a borderline impact on OS was found (p = 0.051).

In our stage I–III material, patients with high expression of miR-126 in both tumor and stroma, show a better 5-year DSS than patients with low expression. Several studies have assessed the association between miR-126-expression and metastatic colorectal cancer (stage IV). Hansen et al. published a study in 2012 looking at the predictive value of miR-126 in patients treated with first line palliative chemotherapy^12^. They found that responders to chemotherapy had a significantly higher median miR-126 expression than non-responders (p < 0.001). Median PFS was 11.5 *vs* 6.0 months (p < 0.001) and median OS 26.2 *vs* 16.8 months (p = 0.002) in high *vs* low expression tumors. The same group showed similar results with increased PFS for patients with high miR-126 expression (p = 0.005) when assessing the clinical outcome of patients with metastatic colorectal cancer treated with chemotherapy combined with Bevacizumab (anti-VEGF-A)^22^. It is, however, questionable to compare data from metastatic patients with those treated with curative intent. However, a study by Ebrahimi et al. showed similar levels of miR-126 expression in primary tumors and metastatic lesions^23^. Caution must be taken when correlating these data, as the results from the metastatic patients also included patients with primary rectal cancer.

One of the strengths of this paper is the unselected study population from Northern Norway with a relatively large number of patients. The study has an extended follow-up and includes relevant clinicopathological variables. Although our clinical database comprises patients operated at least 13 years ago, it still is a representative cohort when comparing to the most contemporary data from the Norwegian cancer registry and colorectal cancer statistics from the United States^1,24^. Median age at diagnosis is in the early 70s in Norway, and approximately 1 in 3 patients presents with regional disease (stage III). In the United States, median age at diagnosis has dropped to 69 years, possibly as a result of colon cancer screening programs. Weaknesses of our study are the retrospective design and the former inadequate histological reports when compared to today’s standards. Vascular infiltration was not routinely assessed in this time-period and neither was possibly relevant mutations (KRAS, NRAS, BRAF) nor MSI-status. However, in Norway, MSI assessment is only conducted in patients below 60 years of age at the time of diagnosis, and the previously mentioned mutations only in the case of metastatic disease. In the patient cohort treated with curative intent, this information is missing for a majority of the patients and was consequently omitted from statistical analyses.

Previous studies assessing the prognostic impact of miR-126 expression in colon cancer were mainly performed using qPCR. In the previously mentioned meta-analysis from 2016, ISH was applied in three studies, qRT-PCR in 14 studies^19^. When using RNA extracts from whole tumors you get a mixture of neoplastic tumor cells and tumor-related stromal cells, and this method does not give information about miR-126 expression in the various tumor compartments. By using in situ hybridization in our study, we are able to precisely identify the miR-126 expression in different compartments and cell types. Assessing miR-expression by in situ hybridization has previously been evaluated in a semi-quantitative manner. By using digital pathology for this, we omit inter-observer variability, and results are likely to be more accurate and reproducible. This is particularly useful when analyzing the intensity of the positive, blue staining of miR-126 in this material.

We found statistically significant correlations between miR-126 expression in stroma and pathological stage and histological grade (p = 0.027 and p = 0.004 respectively). However, the true clinical significance of these findings must be considered uncertain due to the low number of patients with undifferentiated tumors (n = 4) that may have contributed to the statistical significance. Colon cancer cell line experiments were inconclusive on this issue. Li et al. found a correlation between miR-126 expression and histological grade, but not pathological stage^25^. Ebrahimi et al., on the other hand, found a correlation between miR-126 expression and pathological stage, but not with histological grade^23^.

Adjuvant chemotherapy is administered to eradicate potential microscopic disease post surgery. It has been shown to increase 5-year DSS and OS for both stage II and III colon cancer patients^26^. The effect is more pronounced in stage III, and as a consequence, Norwegian patients with stage III colon cancer are routinely offered adjuvant chemotherapy unless they have contraindications. For stage II patients the effect is not as clear-cut^18^. Here, adjuvant chemotherapy is offered on an individual basis to high-risk patients (perforated tumors, pT4 or low number of examined lymph nodes). miR-126 expression may aid the oncologist in treatment-decision-making for stage II colon cancer patients and help select patients likely to benefit from potentially harmful adjuvant chemotherapy. However, before clinical implementation can commence, a standardized assessment of miR-126 must be established and prospective clinical trials conducted.

## Conclusion

We have shown that a high expression of miR-126 is an independent positive prognostic factor for stage I–III colon cancer patients. An improved 5-year DSS was seen for patients with a high expression of miR-126 in both cancer cells and stromal cells. Our results largely confirms the results from previous studies. Further, we complement the knowledge of miR-126 expression in colon cancer by investigating its expression in different tumor compartments and by introducing digital pathology. A potential clinical implication of our findings, may be to use miR-126 expression to select the stage II colon cancer patients most likely to benefit from adjuvant chemotherapy.

## Material and methods

### Study population

**Figure 3:**
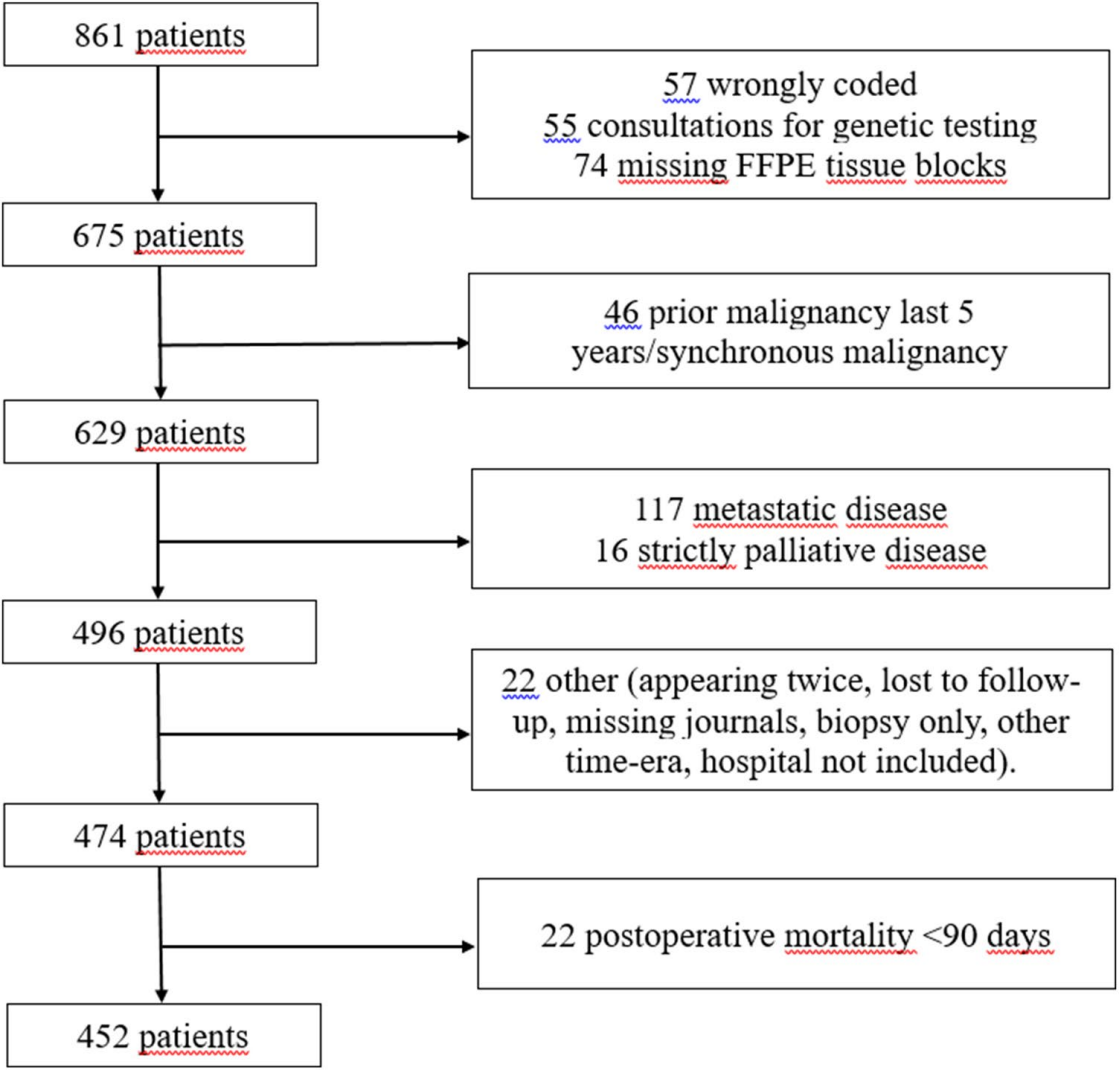
Flowchart of the inclusion of patients in the study.

Patients who underwent radical surgery for colon cancer, in various hospitals in Northern-Norway in the time-period 1998–2007, were eligible for inclusion in this study (Fig. 3). Exclusion criteria were metastatic disease/non-radical surgery, prior malignancy within the last 5 years before colon cancer diagnosis or other synchronous malignancies. A list of patients diagnosed with colon cancer in the time-period was procured from the Department of Clinical Pathology at the University Hospital of North Norway. Of a total of 861 identified patients, 409 patients were excluded due to: metastatic disease at the time of diagnosis (117), tissue-blocks missing or inadequate for TMA construction (79), wrongly coded as colon cancer (57, mainly rectal cancer), genetic testing on patients operated outside of Northern Norway (55), prior malignancies within the last 5 years before colon cancer diagnosis (46), mortality within the first 90 days after surgery (22), surgery for recurrent colon cancer or treated in a strictly palliative setting (18), lost to follow-up (7, three of them tourists), appearing twice on the list (3), surgery at a regional hospital not participating in the study (2), operated before the actual time frame (2) or a missing medical journal (1). Hence, 452 patients were included in this study. Follow-up was completed December 1, 2017.

### Tissue microarray construction

All colon cancer cases were histologically reviewed by two pathologists, and the most representative areas of tumor without necrosis were selected. A 0.6 mm-diameter stylet was used to sample a total of 4 cores securing both tumor tissue and tumor stroma from each included patient. The TMAs were assembled using a tissue-arraying instrument (Beecher Instruments, Silver Springs, MD, USA). The detailed methodology has previously been reported^27^.

### In situ hybridization (ISH)

The method used for in situ hybridization is based on a protocol developed by Jorgensen et al., and adjusted for automatic ISH on the Ventana Discovery-Ultra platform (Roche, Tucson, USA)^28^. Double‐DIG labeled miRCURY LNA detection probes (Exiqon AS, Denmark) were used to visualize miR-126-3p. A scrambled probe and U6 were used as negative and positive controls, respectively. Further, a multi organ TMA section was used as an additional control. Optimizations regarding temperatures, times, and concentrations were done for each probe and reagent.

Slides were baked at 60 °C overnight, and then transferred to the Discovery-Ultra for ISH. Sections were then deparaffinized by heating the slide to 68 °C and incubating for three cycles of 12 min. Antigen retrieval was performed by heating the slides to 95 °C and subsequent treatment with Discovery Cell Conditioning Solution (CC1) for 40 min. The LNA detection probe for miR-126-3p was diluted to a concentration of 2.0 nM, and added manually. The hybridization reaction was carried out at 51 °C for 60 min. Two stringency washes with 2.0X SSC at 51 °C with 8 min incubation before each wash were performed. The slides were incubated with blocking solution for 16 min to block against unspecific bindings. Alkaline phosphatase-conjugated anti DIG (Anti-DIG-AP) was incubated for 20 min for immunologic detection. Substrate enzymatic reactions were carried out with NBT/BCIP for 60 min to give a blue precipitate to detect the miR. The slides were counterstained with Nuclear Fast Red to visualize the nuclei. Slides were dehydrated through an increasing gradient of ethanol solutions and xylene, and mounted with Histokitt mounting medium.

### In situ hybridization scoring/QuPath

TMA slides were digitized using a Pannoramic 250 Flash III (3DHistech, Budapest, Hungary) slide scanner, and processed in QuPath v.0.1.3 (Queen’s University, Belfast, Northern Ireland) in an Ubuntu 20.04 environment. TMA slides were de-arrayed and preprocessed according to Bankhead et al.^29^. Tissue within each TMA core was identified using simple tissue detection and tiled into 10 × 10 μm tiles. Image features were calculated for each tile and used to train a Random Forest model. Each tile was classified as either tumor, stroma, necrosis or other. After classification, tiles were converted into continuous areas and the mean intensity of miR-126 within tumor and stroma were calculated. The scripts used to process the TMAs are included in the supplementary file.

All possible dichotomized cut-offs were evaluated (S2 Fig). For any subsequent analyses the optimal cut-off was chosen.

### Statistical methods

Statistical tests were performed using the statistical packages SPSS version 26.0 or R version 3.6.3. χ^2^ test or Fisher’s exact tests were used to examine the association between molecular marker expression and clinicopathological parameters. DSS (disease-specific survival) was defined as the interval from surgery to the time of colon cancer death. For the univariate analyses, the Kaplan–Meier method was used to visualize associations between molecular marker expression and survival. The log-rank test was used to assess the statistical significance of the differences between the survival curves. Multivariate analyses were performed using a backward conditional Cox regression analysis with a probability for stepwise entry and removal at 0.05 and 0.10, respectively. A p-value < 0.05 was considered statistically significant.

### Ethics declaration

This study was approved by the Regional Committee for Medical and Health Research Ethics North (REK Nord, protocol ID: 2011/2151) and the need for patient consent waived. The reporting of clinicopathological variables, survival data and biomarker expression was conducted in accordance with the REMARK guidelines^30^.

## Supplementary Information

Supplementary Figure Legends.

Supplementary Information.

## Data Availability

Data will be shared upon reasonable request to the corresponding author.
