# Supplementary material for: High expression of microRNA-126 relates to favorable prognosis for colon cancer patients
Source: Sci Rep. 2021 May 5;11:9592. doi: 10.1038/s41598-021-87985-3 (PMC8100289; doi:10.1038/s41598-021-87985-3)
Supplement: Supplementary file 1 — Supplementary Figure Legends. [file 41598_2021_87985_MOESM1_ESM.docx]

**S1 Figure:** Disease-specific survival curves for tumor (A-C) and stromal (D-F), expression of miR-126 in pStage I (A and D), II (B and E) and III (C and F) using the optimal cut-offs for each marker.

**S2 Figure:** All possible dichotomized cut-offs for miR-126, in tumor and stroma, plotted against *P*-values indicating significance of disease-specific survival. The horizontal and vertical lines, represent *P* = 0.05 and median expression values, respectively. The bar-chart represent the number of patients within each 0.1 step increase in expression.
