# Supplementary material for: High expression of microRNA-126 relates to favorable prognosis for colon cancer patients
Source: Sci Rep. 2021 May 5;11:9592. doi: 10.1038/s41598-021-87985-3 (PMC8100289; doi:10.1038/s41598-021-87985-3)
Supplement: Supplementary file 2 — Supplementary Information. [file 41598_2021_87985_MOESM2_ESM.pdf]

High expression of microRNA-126 Relates to Favorable Prognosis for Colon Cancer Patients

Hallgeir Selven[1, 2 *]

Lill-Tove Rasmussen Busund[3, 4]

Sigve Andersen[1, 2]

Roy M. Bremnes[1, 2]

Thomas Karsten Kilvær[1, 2]

1 Department of Oncology, University Hospital of North Norway, Tromso, Norway
2 Department of Clinical Medicine, UiT The Arctic University of Norway, Tromso, Norway
3 Department of Medical Biology, UiT The Arctic University of Norway, Tromso, Norway
4 Department of Clinical Pathology, University Hospital of North Norway, Tromso, Norway

Corresponding author and reprints:
Hallgeir Selven
Department of Oncology, University Hospital of North Norway
9038 Tromso, Norway
Telephone: +47 776 26 765/+47 976 83 966
Fax: +47 776 26 779
E-mail: hallgeir.selven@uit.no

S1 Figure

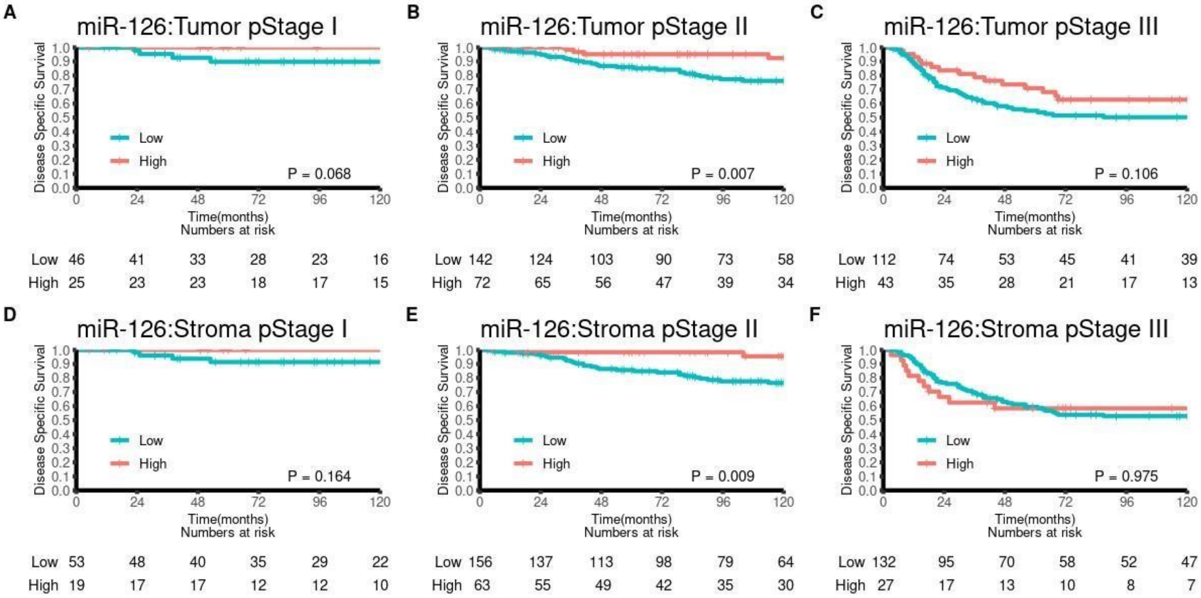
A
miR-126:Tumor pStage I
Disease Specific Survival
Low
High
P = 0.068
Time(months)
Numbers at risk
Low 46 41 33 28 23 16
High 25 23 23 18 17 15
B
miR-126:Tumor pStage II
P = 0.007
Low 142 124 103 90 73 58
High 72 65 56 47 39 34
C
miR-126:Tumor pStage III
P = 0.106
Low 112 74 53 45 41 39
High 43 35 28 21 17 13
D
miR-126:Stroma pStage I
P = 0.164
Low 53 48 40 35 29 22
High 19 17 17 12 12 10
E
miR-126:Stroma pStage II
P = 0.009
Low 156 137 113 98 79 64
High 63 55 49 42 35 30
F
miR-126:Stroma pStage III
P = 0.975
Low 132 95 70 58 52 47
High 27 17 13 10 8 7

S2 Figure

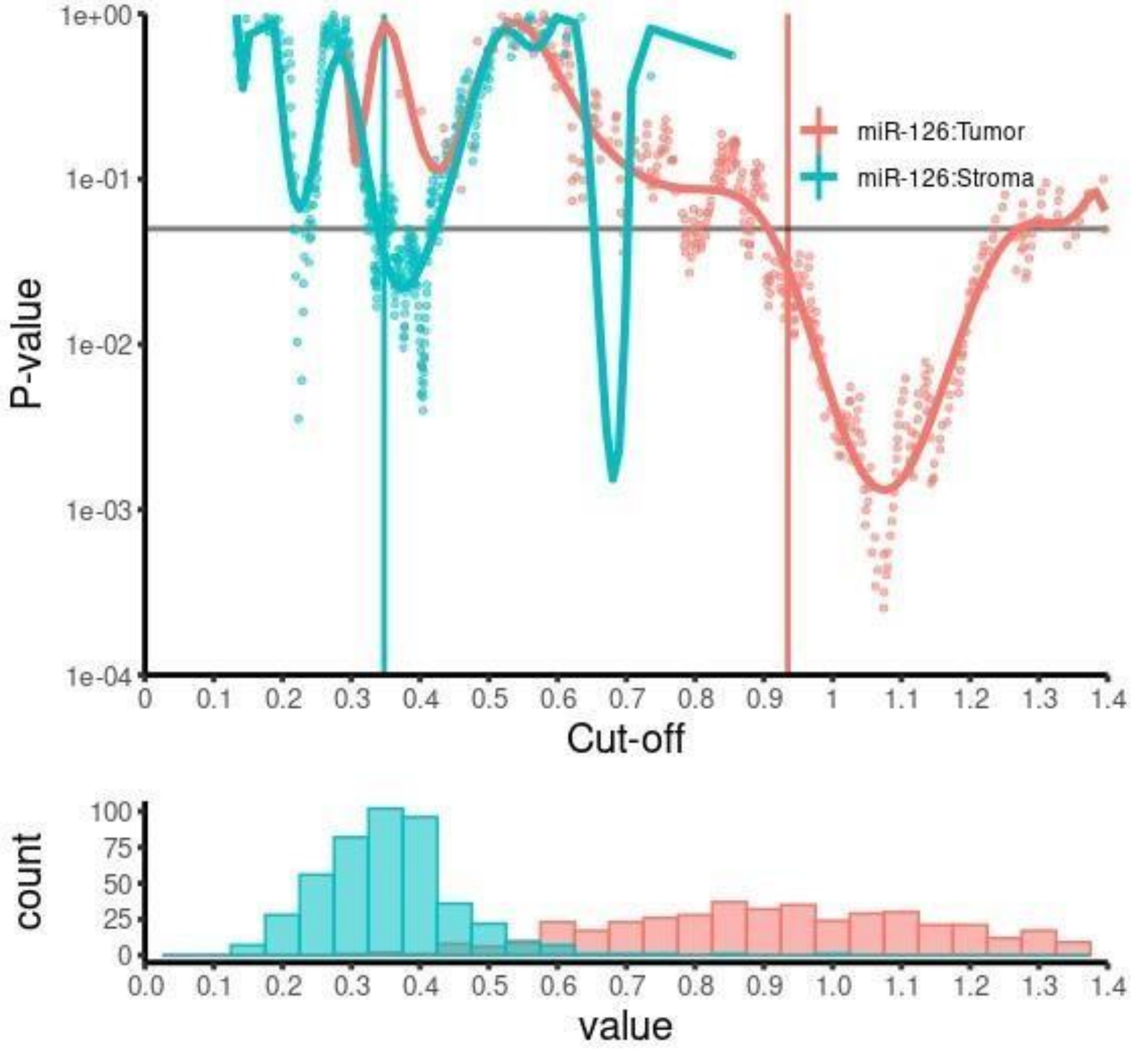

1e+00
1e-01
1e-02
1e-03
1e-04
P-value
miR-126:Tumor
miR-126:Stroma
0 0.1 0.2 0.3 0.4 0.5 0.6 0.7 0.8 0.9 1 1.1 1.2 1.3 1.4
Cut-off
count
100
75
50
25
0
0.0 0.1 0.2 0.3 0.4 0.5 0.6 0.7 0.8 0.9 1.0 1.1 1.2 1.3 1.4
value

```
import qupath.lib.objects.PathAnnotationObject
import qupath.lib.objects.TMACoreObject
import qupath.lib.objects.hierarchy.PathObjectHierarchy

//Preprocessing
setImageType('BRIGHTFIELD_OTHER');
setColorDeconvolutionStains('{"Name" : "H-DAB default", "Stain 1" : "miR-126",
"Values 1" : "0.65123 0.70124 0.2901 ", "Stain 2" : "R", "Values 2" : "0.26896
0.56792 0.7779 ", "Background" : " 255 255 255 "}');
selectTMACores();
runPlugin('qupath.imagej.detect.tissue.SimpleTissueDetection2', '{"threshold": 252,
"requestedPixelSizeMicrons": 1.0,  "minAreaMicrons": 500.0,  "maxHoleAreaMicrons":
500.0,  "darkBackground": false,  "smoothImage": true,  "medianCleanup": true,
"dilateBoundaries": false,  "smoothCoordinates": true,  "excludeOnBoundary": false,
"singleAnnotation": true}');

//Area identification
selectAnnotations();
//Superpixels can be used as an alternative to conventional tiles
runPlugin('qupath.lib.algorithms.TilerPlugin', '{"tileSizeMicrons": 10.0,
"trimToROI": true,  "makeAnnotations": false,  "removeParentAnnotation": false}');
//runPlugin('qupath.imagej.superpixels.SLICSuperpixelsPlugin', '{"sigmaMicrons":
2.0,  "spacingMicrons": 10.0,  "maxIterations": 10,  "regularization": 0.25,
"adaptRegularization": true,  "useDeconvolved": true}');
selectDetections();     runPlugin('qupath.lib.algorithms.IntensityFeaturesPlugin',
'{"pixelSizeMicrons": 2.0,  "region": "ROI",  "tileSizeMicrons": 25.0,  "colorOD":
true,  "colorStain1":
true,  "colorStain2": true,  "colorStain3": false,  "colorRed": true,
"colorGreen": false,  "colorBlue": true,  "colorHue": true,  "colorSaturation":
true,  "colorBrightness": true,  "doMean": true,  "doStdDev": true,  "doMinMax":
true,  "doMedian": true,  "doHaralick": true,  "haralickDistance": 1,
"haralickBins": 32}');
selectAnnotations();
runPlugin('qupath.lib.plugins.objects.SmoothFeaturesPlugin', '{"fwhmMicrons": 25.0,
"smoothWithinClasses": false,  "useLegacyNames": false}');
runPlugin('qupath.lib.plugins.objects.SmoothFeaturesPlugin', '{"fwhmMicrons": 75.0,
"smoothWithinClasses": false,  "useLegacyNames": false}');

//Tidy the annotations
selectAnnotations();
def selected = getSelectedObjects()
removeObjects(selected, true)

//Run classifier and create area annotations
runClassifier('path_to_your_classifier')
selectTMACores();
runPlugin('qupath.lib.analysis.objects.TileClassificationsToAnnotationsPlugin',
'{"pathClass": "All classes",  "deleteTiles": true,  "clearAnnotations": false,
"splitAnnotations": false}'); selectAnnotations();
runPlugin('qupath.lib.algorithms.IntensityFeaturesPlugin', '{"pixelSizeMicrons":
2.0,  "region": "ROI",  "tileSizeMicrons": 25.0,  "colorOD": false,  "colorStain1":
true,    "colorStain2":  false,    "colorStain3":  false,    "colorRed":  false,
"colorGreen": false,  "colorBlue": false,  "colorHue": false,  "colorSaturation":
false,  "colorBrightness": false,  "doMean": true,  "doStdDev": true,  "doMinMax":
```

```
true,    "doMedian":  true,    "doHaralick":  false,    "haralickDistance":  1,
"haralickBins": 32}');

//Add measurements from area annotations to the TMACoreObject
def addMeasurements(PathObjectHierarchy hierarchy, TMACoreObject core, pixelWidth,
pixelHeight) {
    def annotations = hierarchy.getDescendantObjects(core, null,
PathAnnotationObject)

    //Check if core has annotation
if (annotations.size() > 0) {

        //check if each annotation is of interest, and if so, keep track of the
positive and negative cells within         annotations.each { annotation ->
annotationName = annotation.getPathClass().getName()
            if (annotation.getROI().getScaledArea(pixelWidth, pixelHeight) >
MIN_AREA_MICRONS) {
                def measures = annotation.getMeasurementList()
measures.each {
                    def measureName = it.getName()tokenize()[5,6].join()
def measure = it.getValue()
                    core.getMeasurementList().putMeasurement("${annotationName} $
{measureName}", measure)
                }
            }
        }
    }else{
    core.setMissing(true)
    }
}
MIN_AREA_MICRONS = 40000
def server = getCurrentImageData().getServer()
double pixelWidth = server.getPixelWidthMicrons()
double pixelHeight = server.getPixelHeightMicrons()
def hierarchy = getCurrentHierarchy()
def cores = hierarchy.getTMAGrid().getTMACoreList()
cores.each {
    addMeasurements(hierarchy, it, pixelWidth, pixelHeight)
}
fireHierarchyUpdate()
println("Finished")
```
